# Rheumatoid Arthritis Patients With Circulating Extracellular Vesicles Positive for IgM Rheumatoid Factor Have Higher Disease Activity

**DOI:** 10.3389/fimmu.2018.02388

**Published:** 2018-10-29

**Authors:** Onno J. Arntz, Bartijn C. H. Pieters, Rogier M. Thurlings, Mark H. Wenink, Peter L. E. M. van Lent, Marije I. Koenders, Frank H. J. van den Hoogen, Peter M. van der Kraan, Fons A. J. van de Loo

**Affiliations:** ^1^Department of Rheumatology, Radboud University Medical Center, Nijmegen, Netherlands; ^2^Department of Rheumatology, Sint Maartenskliniek, Nijmegen, Netherlands

**Keywords:** rheumatoid arthritis, rheumatoid factor IgM, extracellular vesicles, disease activity score 28, C-reactive protein, ESR, plasma

## Abstract

Rheumatoid arthritis (RA) is an autoimmune inflammatory disease that mainly affects synovial joints. Validated laboratory parameters for RA diagnosis are higher blood levels of rheumatoid factor IgM (IgM-RF), anti-citrullinated protein autoantibodies (ACPA), C-reactive protein (CRP) levels and erythrocyte sedimentation rate (ESR). Clinical parameters used are the number of tender (TJC) and swollen joints (SJC) and the global patient visual analog score (VAS). To determine disease remission in patients a disease activity score (DAS28) can be calculated based on SJC, TJC, VAS, and ESR (or alternatively CRP). However, subtle and better predictive changes to follow treatment responses in individual patients cannot be measured by the above mentioned parameters nor by measuring cytokine levels in blood. As extracellular vesicles (EVs) play a role in intercellular communication and carry a multitude of signals we set out to determine their value as a biomarker for disease activity. EVs were isolated from platelet-free plasma of 41 RA patients and 24 healthy controls (HC) by size exclusion chromatography (SEC). We quantified the particle and protein concentration, using NanoSight particle tracking analysis and micro-BCA, respectively, and observed no differences between RA patients and HC. In plasma of 28 out of 41 RA patients IgM-RF was detectable by ELISA, and in 13 out of these 28 seropositive RA patients (RF^+^RA) IgM-RF was also detected on their isolated pEVs (IgM-RF^+^). In seronegative RA patients (RF^−^RA) we did not find any RF present on pEVs. When comparing disease parameters we found no differences between RF^+^RA and RF^−^RA patients, except for increased ESR levels in RF^+^RA patients. However, RF^+^RA patients with IgM-RF^+^ pEVs showed significantly higher levels of CRP and ESR and also VAS and DAS28 were significantly increased compared to RA^+^ patients without IgM-RF^+^ pEVs. This study shows for the first time the presence of IgM-RF on pEVs in a proportion of RF^+^RA patients with a higher disease activity.

## Introduction

Rheumatoid arthritis is an autoimmune disease in which the body's immune system mistakenly attacks the synovial joints leading to joint inflammation as a hallmark feature of this disease (1). The diagnosis of RA relies on physical examination and laboratory blood testing. Rheumatoid factor (RF) is found in 50% of early RA patients rising to 90% at advanced stages of disease (2). Seropositive RA patients (RF^+^RA) have detectable RF in their circulation and it has been shown that high RF levels are predictors of more severe disease forms (3). The pathogenesis of RA may be different between RF^+^RA and RF^−^RA patients with the later often reported as less severe although studies are conflicting (4). RF contribute to the disease process of RA by a mechanism where large immune complexes are formed and complement activation is induced (5). Cardiovascular disease and organ involvement such as the lungs, heart, and eyes are well-recognized complications occurring primarily in RF^+^RA patients (6).

To determine the disease activity in all RA patients a formula has been developed (DAS28) by our department that includes the physical examination of 28 joints for tenderness and swelling, the erythrocyte sedimentation rate (or C-reactive protein levels instead) and a patient global visual analog score (of pain or global health) (7). DAS28 is a good tool to define remission in established RA but has limited value in monitoring disease processes and responses. It is well-recognized that numerous cytokines play an important role in the local and systemic inflammatory response in RA and although targeting these cytokines using biological is therapeutically successful in many RA patients their usefulness as biomarkers to monitor disease activity and treatment response is poor, even a combination of 12 cytokines in the multi-biomarker disease activity (MBDA) score has limited predictive value (8). Until now, most research on rheumatoid arthritis has focused on cytokines as main effectors in disease progression however, cell-cell communication involves a much broader scope of responses including proliferation, apoptosis and migration. In terms of communication a cell can release additionally extracellular vesicles (EVs) and recently an important role of these EVs has been postulated as important communicators between resident and inflammatory cells (9).

EVs play a regulatory role in immunity during health and disease (10, 11) and are suggested to be involved in auto-immune disease (12). They are released from cells and are detectable in body fluids such as blood, synovial fluid, urine and breast milk (13). They contain numerous proteins (including cytokines), lipids (prostaglandins), RNA, DNA, and sometimes even cell organelles such as mitochondria (14). Three subtypes of EVs have been described: apoptotic bodies (1–10 μm), microvesicles (100–1000 nm), and exosomes (30–100 nm), which are classified based on their origin, programmed cell disintegration, plasma membrane outward budding, and release via multivesicular bodies, respectively (15). Interestingly, specific membrane proteins are enriched in exosomes in a cell-type dependent fashion (13, 16). Additionally, preliminary data suggest that exosome content differs between individual people. This enrichment of specific proteins in exosomes compared to those that are found in the cytoplasm of the donor cell could mean these vesicles play some distinctive function (16).

It is known that B-cells also release EVs that contain the B-cell receptor on their surface which is an immunoglobulin that binds and presents antigens to T-cells (17). The presence of IgM-RF in RF^+^RA patients clearly points to a role of B-cells and plasma cells in the pathogenesis of this disease (18). A B-cell depleting strategy by a monoclonal anti-CD20 antibody (Rituximab) is a highly effective therapy in RA patients (19). Therefore, we investigated the presence of IgM-RF on EVs and its relation to the activity of RA determined by laboratory and clinical parameters.

## Methods

### Blood donors

Blood was obtained from 41 RA patients fulfilling ACR/EULAR classification (20), of which 28 patients had a IgM-RF titer > 10 IU/ml (RF^+^RA). Tender (TJC) and swollen joints (SJC) were assessed by physicians and the global patient visual analog score (VAS) was determined by the patient, where a point was set on a 100 mm line as a reflexion of their disease feeling after which the distance to the point was measured (0 = no disease, 100 = worst disease feeling).

ESR and CRP levels were determined by standard laboratory blood tests in our hospital. Disease activity score (DAS28) was calculated based on SJC, TJC, VAS, and ESR. An overview of the rheumatic arthritis disease status and medication of RF^+^RA and RF^−^RA patients are shown in Table 1. As age matched controls, blood from healthy controls (HC) were obtained from the blood transfusion department (Sanquin, Nijmegen, The Netherlands). All donors provided informed consent under institutional ethics committee approved protocols.

**Table 1:** Clinical characteristics of RA patients at time of blood collection.

		**Rheumatoid arthritis disease status**				
		**RF**^**−**^**RA (*****n*** = **13)**		**RF**^**+**^**RA (*****n*** = **28)**	
		**Mean**	**Count**	**Mean**	**Count**	***P*-value**
Age in years		57 [39–75]		61 [26–85]		0.256
Gender	male		4 (30.8%)		6 (21.4%)
	female		9 (69.2%)		22 (78.6%)
DAS-28 score		3.33 [1.53–5.56]		4.13 [1.60–6.53]		0.101
IgM-RF	-		13 (100%)		-
	+		-		28 (100%)
ESR (mm/hr)		15.5 [2–44]		30.6 [2–104]		0.016
CRP (mg/l)		9.8 [1–58]		15.4 [7–76]		0.140
VAS		55.3 [0–78]		46.7 [0–100]		0.194
TJC		2.6 [0–8]		4.8 [0–22]		0.075
SJC		2.2 [0–10]		4.1 [0–16]		0.051
DMARDs use			11 (84.6%)		20 (71.4%)
Solumedrol use			4 (30.8%)		18 (64.3%)
Analgesics use			10 (76.0%)		22 (78.6%)
Biological use			2 (15.4%)		6 (21.4%)

### Blood sample preparation

Blood samples were taken in ethylenediaminetetraacetic acid tubes (BD, Plymouth, UK) and within 1 h centrifuged for 10 min at 1690 g by 4°C to obtain plasma. Plasma was centrifuged at 10,000 g for 30 min by 4°C to obtain platelet free plasma (pfp). Thereafter the supernatant was passed through a 0.22 μm filter (Whatmann) and aliquoted. Aliquots were stored at −80°C until EV isolation.

### Plasma EV isolation

pEVs were isolated by size exclusion chromatography (SEC) using the protocol described by Lobb et al. (21). In short, a sterile column was prepared for SEC using a 10 ml syringe stacked with sepharose CL-2B (Pharmacia, Uppsala, Sweden). After washing the column with phosphate buffered saline (PBS) containing 0.32% citrate (pH 7.4, autoclaved), 0.5 ml of pfp was loaded and eluted using PBS/0.32% citrate buffer. 1 ml eluate fractions were collected and fraction 5 containing the EVs was stored at 4°C for further use.

### Protein measurement

The amount of protein was measured using a Micro BCA Protein assay kit following the protocol provided by the manufacturer (Thermoscientific, Rockford, USA). pEV samples were diluted 10, 20, 40, and 80 times in NaCl 0.9% and after 2 h incubation at 37°C absorbance was measured using the BioRad iMark microplate reader.

### Nanoparticle tracking analysis

Vesicle size distribution was estimated by the Brownian motion of particles using a NanoSight NS300 (Sysmex, Etten-Leur, The Netherlands) with Nanoparticle Tracking Analysis 3.2 software (NanoSight, Amesbury, UK). Vesicles were diluted in PBS, till an optimal concentration for reliable analysis was reached (20–80 particles per frame). Each sample was measured for 60 s (in duplicates), using the following software settings: flow rate 50, camera level 10 and detection threshold 5.

### Transmission electron microscopy

Fraction 5 of SEC was washed using Vivaspin-2 columns (Sartorius Stedim Biotech GmbH, Goettingen, Germany) to remove salts from the solution and 3 μl EVs in deionized water was placed on a nickel grid and allowed to dry to air for 45 min. The grids were then washed by transferring them onto several drops of deionized water. Negative contrast staining was performed by incubating the grids on top of drops of 6% uranyl acetate. Excess fluid was removed and the grids were allowed to dry before examination in a JEM1200 transmission electron microscope (Jeol, The Netherlands).

### Sucrose gradient

The density of isolated pEVs was determined by sucrose gradient described by Chiou (22). In short, 100 μl SEC isolated pEVs was mixed with 1 ml 70% sucrose (Sigma). Thereafter, a sucrose gradient was layered on top of the 70% in a centrifuge tube (14 × 19 mm; Beckman Instruments Inc. USA) and spun for 18 h in a SW 40 Ti rotor (100,000 g at 4°C). After centrifugation, 1 ml fractions were taken and washed with PBS. Finally, EVs were pelleted at 166,000 g for 90 min and were taken up in 100 ul PBS. Particle concentration was measured by NTA.

### IgM-RF detection

IgM-RF was detected by ELISA. In short, in a 96 wells plate aggregated human IgG (Sigma) was coated. After washing, pEVs were added and incubated for 90 min. Wells were washed twice, thereafter HRP-labeled Goat-anti-IgM F(ab')_2_ fragment (BioMP) was added and incubated for 90 min. Following a third washing step TMB was added to bind HRP. By addition of acid, the enzyme reaction was stopped and plate was measured at 450 nm. IgM levels were calculated using a standard curve. RA patients with an IgM-RF titer > 10 IU/ml in plasma were scored as RF^+^ at the clinical lab. For pEVs IgM-RF levels > 2 IU/ml were regarded as IgM-RF^+^ pEVs in our study.

### Detection of labeled pEVs bound to protein L beads

Based on the protocol previously described (23) pEVs were diluted in PBS before addition of 300 μl of Diluent C and 1 μl PKH-26 (Sigma). The samples were gently mixed for 2 min before adding 500 μl 1% BSA to stop the membrane staining and were loaded onto 300 kDa Vivaspin filters (Sartorius Stedim Biotech GmbH, Goettingen, Germany). After centrifuged at 4,000 g samples were washed three times with 2 ml PBS and taken up in 500 μl PBS. Labeled pEVs were bound to immobilized Protein L magnetic beads (Thermoscientific, Rockford, USA). After short incubation in a magnet the unbound pEVs were collected. After washing three times with PBS + 0.1%BSA pEVs bound to Protein L magnetic beads were collected. Fluorescence of Protein-L bound and unbound pEVs was measured on a fluorometer (Clariostar, BMG).

### Detection of IgG binding to pEVs

IgG binding to IgM-RF^+^ pEVs and protein-L unbound IgM-RF^+^pEVs was measured by bead-assisted flowcytometry (FC). pEVs were coupled to antiCD63 magnetic beads (Thermo Fisher Scientific) for 3 h at RT. Unbound pEVs were removed by 3 washing steps. Thereafter CD63^+^ pEVs were incubated with IgG-PE (eBioscience) for 1 h and after 3 washing steps coupled IgG-PE was measured by FC.

### Statistical analysis

All data are expressed as mean ± SD. Data were compared using two tailed Mann-Whitney U-test. Values of *P* < 0.05 were considered to indicate statistical significance. Correlations were represented by the Pearson correlation coefficient (r) and their *p*-value. All statistical analyses were performed using GraphPad Prism 5.01 (GraphPad Software, La Jolla, CA).

## Results

### Characterization of pEVs

**Figure 1:**
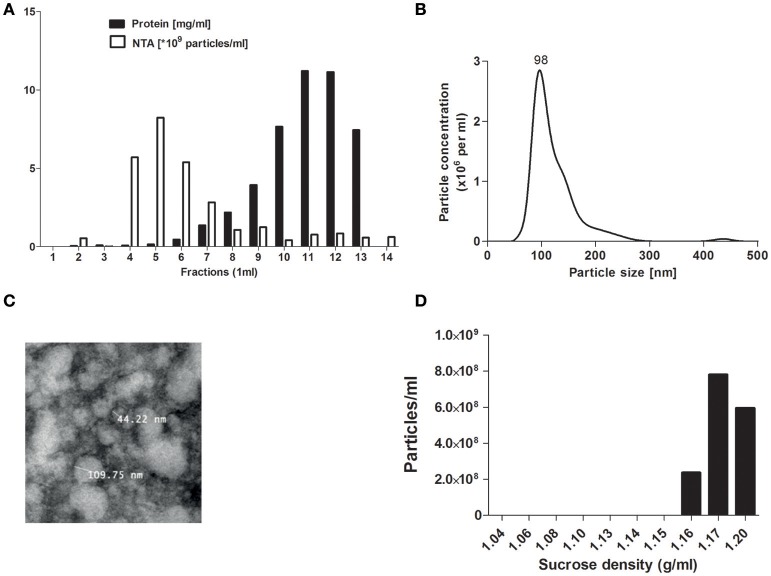
pEV isolation by SEC. Platelet free plasma components were separated by size exclusion chromatography (SEC). For each 1 ml fraction, protein and particle concentrations were measured by micro-BCA protein assay and Nanosight tracking analysis (NTA) **(A)**. Particle size from SEC fraction 5 was analyzed by NTA **(B)** and TEM **(C)**. Density of pEVs was determined by use of a sucrose gradient and particles of each density fraction were measured by NTA **(D)**.

To separate EVs from plasma proteins SEC isolation was used as described before (21). Collection of eluent showed that fraction 5 displayed the highest particle concentration (detected by NTA) while in fractions 6 and above high protein levels were detected (measured by BCA measurement) (Figure 1A). The particle size of SEC eluted fraction 5 was around 100 nm as determined by NTA (Figure 1B), in line with the observed size visualized by TEM (Figure 1C). For all further studies SEC eluent fraction 5 was used. The main density of the isolated pEVs was 1.17 g/ml (sucrose density) (Figure 1D), which confirmed the presence of exosomes.

### Comparison of pEVs from RA patients and healthy subjects

**Figure 2:**
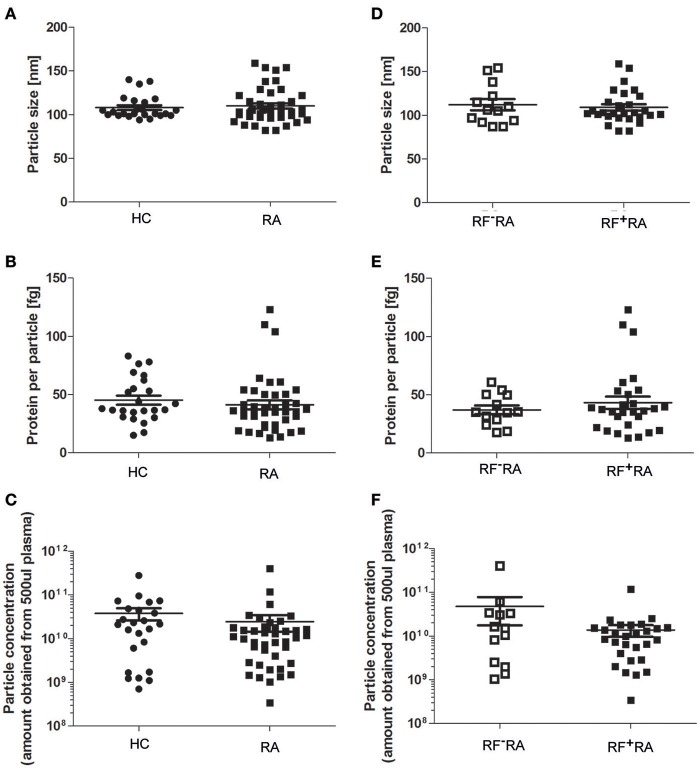
Characterization of pEVs from healthy controls and RA patients. Mode particle size **(A)**, protein content per particle **(B)**, and amount of particles **(C)** from pEVs isolated from 500 μl pfp of HC (*n* = 24) or RA patients (*n* = 41) were measured by NTA and micro-BCA protein assay. Next, RA patients were divided in 2 groups based on the presence of IgM-RF (RF^+^RA; *n* = 28 and RF^−^RA; *n* = 13). Particle size **(D)**, protein per particle **(E)** and amount of particles **(F)** of isolated pEVs are shown measured by NTA and micro-BCA protein assay.

**Figure 3:**
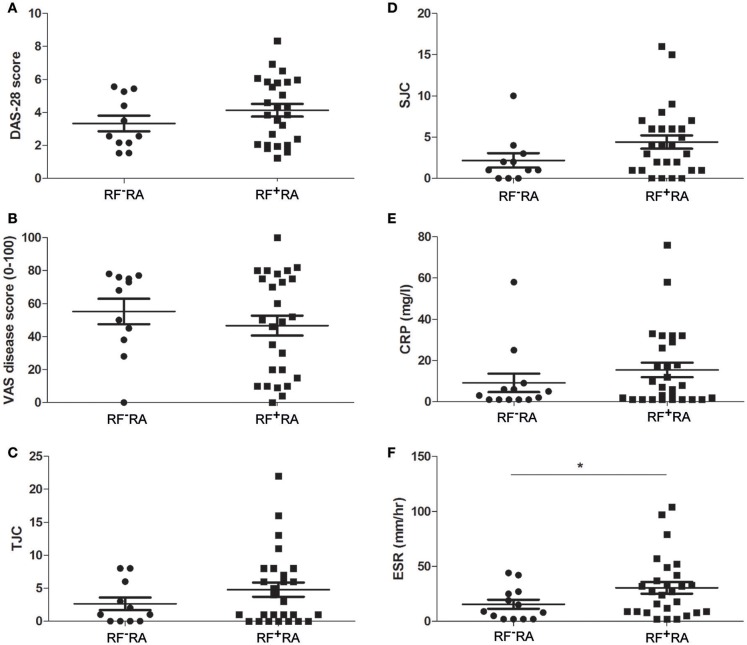
Clinical parameters related to the presence of IgM-RF in blood. Based on the presence of IgM-RF in blood the RA patients were divided 2 groups, RF^+^RA and RF^−^RA patients. Clinical parameters were obtained at time of blood donation. DAS28 **(A)**, VAS disease activity **(B)**, TJC **(C)**, and SJC **(D)**, and CRP and ESR levels **(E,F)** of RF^+^RA and RF^−^RA patients are shown. Statistically significant differences were determined by Mann-Whitney test, ^*^p < 0.05.

When comparing pEVs isolated from RA patients and HC, we observed no differences in particle size (Figure 2A), protein content (Figure 2B) and particle concentration (Figure 2C) (115 nm, 49fg, 3.5 × 10^10^ and 108 nm, 45fg, 3.8 × 10^10^, respectively). In addition, between RF^−^RA and RF^+^RA patients, particle size (Figure 2D), protein content per particle (Figure 2E) and amount of particles (Figure 2F) of pEVs were not statistically different (116 nm, 39fg, 5.410^10^ and 114 nm, 58fg, 1.8 × 10^10^, respectively). The RF^−^RA and RF^+^RA patient groups did not statistically differ in terms of demographics (Table 1), clinical parameters (DAS28, VAS, TJC and SJC) and CRP (Figures 3A–E, respectively) except for the erythrocyte sedimentation rate (Figure 3F) which was higher in the RF^+^RA group.

### Detecting RF on pEVs in RA patients

**Figure 4:**
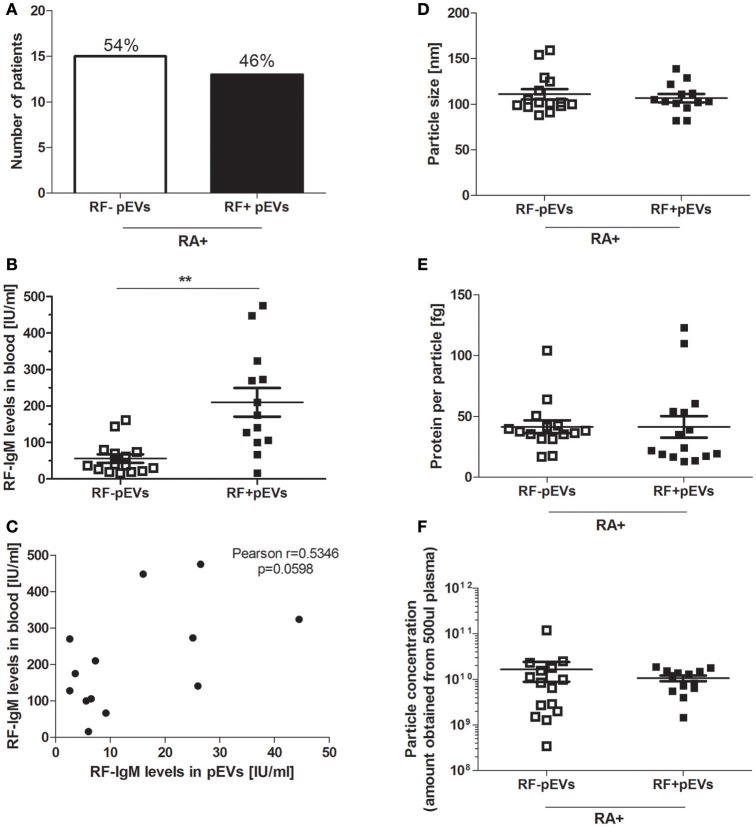
IgM-RF detection in fraction 5 of SEC separated platelet free plasma. IgM-RF levels on pEVs isolated from all 28 RF^+^RA patients were measured by ELISA and percentage of RF^+^RA patients with or without IgM-RF^+^ pEVs are shown **(A)**. From these 2 subdivided groups IgM-RF blood levels are shown individually per patient **(B)**. Correlation of IgM-RF levels on pEVs and levels in platelet free plasma (pfp) of RF^+^RA patients with IgM-RF^+^ pEVs is shown **(C)**. Pearson correlation coefficient (r) and their *p*-value is shown. Mode particle size **(D)**, protein content per particle **(E)** and amount of particles **(F)** from pEVs of these subdivided groups (RF^+^RA patients with IgM-RF^+^ pEVs or IgM-RF^−^ pEVs) are shown, measured by NTA and micro-BCA protein assay. Statistically significant differences were determined by Mann–Whitney test, ^**^p < 0.01.

To investigate whether IgM-RF, as detected in plasma of RF^+^RA patients by ELISA, could also be detected in fraction 5 of the SEC isolated pEVs, IgM-RF ELISA was performed and in 46% (13 out of 28 RF^+^RA patients) IgM-RF was detectable (Figure 4A) while no IgM-RF was found in SEC eluent fraction 5 of RF^−^RA patients (data not shown). IgM-RF levels in pfp were significantly enhanced in RF^+^RA patients with IgM-RF^+^ pEVs, although a high variability was observed in this group (Figure 4B). This high variability and the result that levels of IgM-RF as determined in SEC fraction 5 did not correlate with the plasma levels of IgM-RF (Figure 4C) excludes the co-isolation of plasma RF protein in SEC fraction 5. No differences in particle size, particle concentration and protein concentration per particle were observed between RF^+^RA patients with or without IgM-RF on their pEVs (Figures 4D–F respectively).

### Confirming the presence of IgM-RF on pEVs

**Figure 5:**
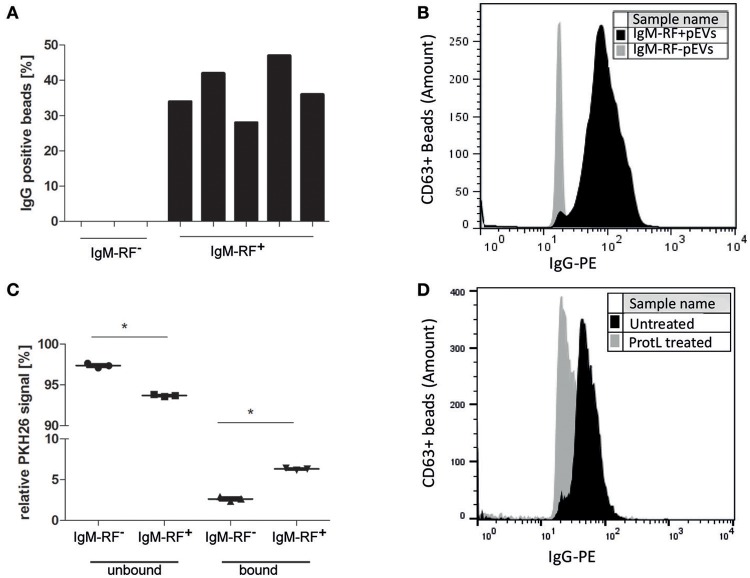
Antibody (IgG) binding to IgM-RF containing pEVs. SEC fraction 5 of 8 RA patients were incubated to anti-CD63 beads to specifically immobilize the EVs to the beads. Thereafter, the beads were incubated with human IgG-PE and analyzed by FC. IgG binding to pEVs coupled to anti-CD63 magnetic beads was only found in the RF^+^RA patients with IgM-RF^+^ pEVs **(A)**. A representative FC plot is shown of a RF^+^RA patients with IgM-RF^+^ pEVs (black) and RF^+^RA patients with IgM-RF^−^ pEVs (gray) **(B)**. **(A,B)** showed a IgG binding factor on pEVs. Next pEVs were stained by PKH26 and incubated with protein-L beads. The level of bound and unbound PKH26-stained pEVs was measured by fluorometer **(C)**. Reduced IgG-PE staining was observed on Protein L unbound IgM-RF^+^ pEVs bound to CD63 magnetic beads (black) as measured by FC **(D)**. Statistically significant differences were determined by Mann–Whitney test, ^*^p < 0.05.

RF are autoantibodies directed against the Fc-tail of immunoglobulin G. To study the IgG binding to IgM-RF^+^ pEVs, we coupled pEVs isolated from 8 RF^+^RA patients to anti-CD63 beads, incubated them with Phycoerythrin (PE)-conjugated IgG and analyzed IgG binding using FC. IgG-PE was bound to pEVs in the same RF^+^RA patients in which IgM-RF was detected on EVs by ELISA (Figure 5A). A representative FC plot of an RA patient with IgM-RF^+^ pEVs is shown (Figure 5B). To investigate the amount of IgM-RF^+^ pEVs in the total isolated pEVs, pEVs were labeled with the fluorescent membrane staining PKH26, thereafter incubated with protein-L beads. Fluorescence was measured in the protein-L bound- and unbound fractions. On average 3,6% more fluorescence signal was detected in the protein-L bound fraction of the IgM-RF^+^ pEVs compared with the IgM-RF^−^ pEVs (Figure 5C). Detection of IgG binding to protein-L unbound pEVs showed a decreased PE signal indicating the presence of IgM-RF on the pEVs (Figure 5D). These two different approaches confirmed that the IgM-RF^+^ particles are actually EVs because they have a lipid bilayer and carry the exosome marker CD63.

### RF^+^RA patients with IgM-RF containing pEVs showed more severe disease

**Figure 6:**
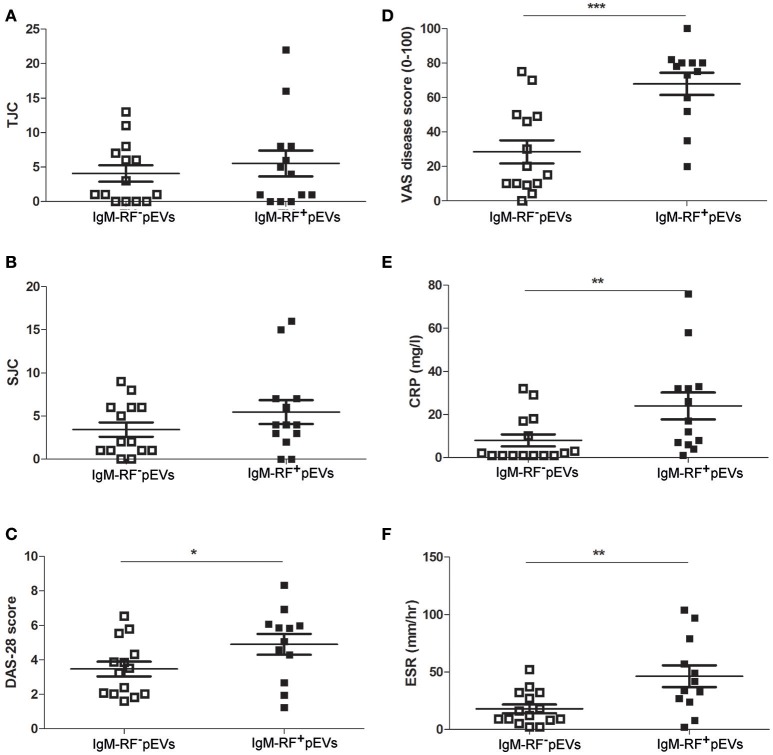
Clinical parameters related to IgM-RF containing pEVs. Clinical parameters were obtained at time of blood donation. RF^+^RA patients were dived in 2 subgroups based on the presence of IgM-RF^+^ pEVs and IgM-RF^−^ pEVs in their circulation. TJC **(A)**, SJC **(B)**, DAS28 **(C)**, VAS disease activity **(D)**, CRP and ESR levels **(E,F)** of these 2 subgroups are shown. Statistically significant differences were determined by Mann-Whitney test, ^*^p < 0.05, ^**^p < 0.01 and ^***^p < 0.001.

An overview of clinical data obtained from RF^+^RA patients stratified in two groups based on presence of IgM-RF on their pEVs is shown in Table 2. As IgM-RF and EVs are thought to be implicated in the disease process, we divided the RF^+^RA patients in 2 subgroups based on the presence or absence of IgM-RF on their pEVs (IgM-RF^+^ pEVs and IgM-RF^−^ pEVs). After analyzing the coupling of the obtained clinical parameters to these two subgroups we observed no differences on TJC and SJC (Figures 6A,B). However DAS28, VAS disease activity, CRP and ESR levels were significantly enhanced in patients with IgM-RF^+^ pEVs (Figures 6C–F, respectively), suggesting the presence of IgM-RF on pEVs to be a novel biomarker for disease activity in RA patients.

**Table 2:** Clinical data from RF+RA patients stratified in two groups based on presence of IgM-RF on their pEVs.

		**Rheumatoid arthritis disease status of RF**^**+**^**RA patients**				
		**IgM-RF**^**−**^**pEVs (*****n*** = **15)**		**IgM-RF**^**+**^ **pEVs (*****n*** = **13)**	
		**Mean**	**Count**	**Mean**	**Count**	***P*-value**
Age in years		59 [26–85]		62 [31–84]		0.320
Gender	male		2 (13.3%)		4 (30.8%)
	female		13 (86.7%)		9 (69.2%)
DAS-28 score		3.47 [1.60–6.53]		4.90 [1.24–8.33]		0.033
IgM-RF	-		-		-
	+		15 (100%)		13 (100%)
ESR (mm/hr)		18.0 [2–52]		46.3 [2–104]		0.007
CRP (mg/l)		8.0 [1–32]		24.0 [1–76]		0.016
VAS		28.4 [0–75]		67.9 [20–100]		0.0001
TJC		4.1 [0–13]		5.5 [0–22]		0.258
SJC		3.4 [0–9]		5.5 [0–16]		0.175
DMARDs use			13 (86.7%)		7 (53.8%)
Solumedrol use			8 (53.3%)		10 (76.9%)
Analgesics use			11 (73.3%)		11 (84.6%)
Biological use			2 (13.3%)		4 (30.8%)

## Discussion

In this study plasma EVs isolated from healthy controls an RA patients did not show different characteristics in all parameters studied, however in seropositive RA patients we have identified a subgroup expressing IgM-RF on their pEVs and these patients showing significantly higher disease activity.

We set out to study the putative differences between EVs from RA patients and healthy controls as well as between serological different RA patient groups. Others found that the number of circulating EVs is enhanced in RA patients (24) but we could not confirm this result in our study. The total particle concentration of pEVs obtained was not different between RA patients and HC. We also showed that the protein content and particle size of pEVs isolated from RA patients were comparable to HC although others reported that the protein content of EVs from patients with RA is altered (25). The coagulation cascade in serum can lead to massive EV release by thrombocytes as well as by storage at −80°C (26) and the observed differences could be explained by the fact that platelet counts are increased in RA patients (27).

Our study shows that IgM-RF is present in the pEVs isolated after SEC separation in a subset of RF^+^RA patients, and not in RF^−^RA patients nor healthy controls. The fact that IgM-RF levels in the SEC isolated pEVs did not correlate to levels detected in plasma makes it plausible that IgM-RF is bound to pEVs. Furthermore, the presence of a lipid bilayer (PKH26 staining) and binding to anti-CD63 confirmed that these IgM-RF particles makes them true extracellular vesicles.

ESR was enhanced in RF^+^RA patients compared to RF^−^RA patients while other clinical parameters were not statistically different. In this study we found that RF^+^RA patients with detectable IgM-RF on their pEVs showed significantly higher ESR compared to RF^+^RA patients without IgM-RF on their pEVs and also CRP levels were statistically enhanced. CRP is a sensitive index for RA disease activity and changes in CRP can predict treatment response of patients (28). Interestingly, the objective joint scores (TJC and SJC), which are considered the most robust reflector of disease activity, did not differ between these subgroups while the VAS (global assessment of disease activity) was clearly increased. The VAS is a more subjective index of patients general health of which pain and probably other symptoms and manifestations of RA are reflected. The disparity between global assessment of disease activity and the number of tender and swollen joints is striking, however appears to be in line with clinical literature in which (changes in) global VAS are poorly explained (29). Additionally, the DAS28 was significantly enhanced in RA patients with IgM-RF on their pEVs suggesting that DAS28, VAS, ESR, and CRP levels reflect more the systemic disease component/phenotype of RA in patients.

The enhanced levels of ESR, CRP and elevated VAS and DAS28 we found in RA patients with IgM-RF^+^ pEVs may suggest that these pEVs contributes to disease pathophysiology in these patients. Autoantibodies like IgM-RF are very efficient activators of the complement by a reaction with IgG (30). This activation leads to the production of inflammatory cytokines in synovial cells which can induce inflammation, cartilage damage and bone erosion (1). In RA synovium Sato et al. have found a more complement-dependency of inflammation with IgM-RF and IgG3 than with other IgG subclasses suggested a dominant role of IgG3 in RA (31), both IgM and IgG3 are the most efficient of the human immunoglobulin (sub)classes in activating complement. Saunderson et al. described the presence of IgG on exosomes derived from B-cells (32). It is plausible that IgM-RF is bound to IgG present on pEVs or that it recognizes IgG autoantibodies that bind to antigens on EVs. Another possibility is that the IgM-RF^+^ pEVs originate from autoreactive B-cells and as B-cells play an important role in RA (18) there could be a of changes in BCR repertoire, or enhanced B-cell activation in this subgroup of RA patients with a more active disease. In that case the IgM-RF^+^ pEVs could reflect changes in pre-B-cell immunity and disease activity more rapidly than changes in circulating levels of “free” IgM-RF, which reflects plasma activity. The reported short circulation half-life of EVs in hours could make the IgM-RF^+^ pEVs more responsive to changes in disease activity than free IgM-RF having a half-life of 5 days in the circulation (33).

For future study, it would be interesting to measure the effect of the B-cell depletion therapy by Rituximab on IgM-RF^+^ pEVs and investigate whether changes in IgM-RF^+^ pEVs levels reflect the drop in B-cells and the clinical response to this treatment. In that case, measuring IgM-RF^+^ pEVs levels might be a way to determine the efficacy of B-cell depletion therapy. Furthermore, longitudinal studies may reveal whether we are dealing with a unique subpopulation of RA patients or that the IgM-RF^+^ pEVs merely reflects the disease state at the moment of blood sampling. The IgM-RF^+^ pEVs levels could be a reflection of a combination of inflammation and autoreactive B-cells activation.

It is plausible that Fc-receptors are activated by immune-complexes formed by IgM-RF^+^ pEVs. Therefore, further research is needed to investigate the added value of the presence of EVs in the immune-complex induced Fc-receptor signaling. To study their functional effect on immune cells requires the use of pure IgM-RF^+^ pEVs and a recent described technique would be suitable to separate IgM-RF^+^ pEVs from the total pEVs population isolated from RA patients by advanced imaging flow cytometry (34).

In conclusion, the present study shows for the first time that in a subset of seropositive RA patients IgM-RF is present on pEVs and this is related to higher disease activity. Our discovery sheds new light on the disparity between global assessment of disease activity and tender and swollen joints seemingly uncovering a potential biological factor in the “subjective” measure of global disease activity.

## Author contributions

OA, BP, RT, and FvdL participated in the design of the study. OA and BP contributed in the experimental methods. OA and BP performed data analysis. OA, BP, FvdL wrote the manuscript. OA, BP, RT, MW, PvL, PvdK, MK, FvdH, and FvdL contributed to discussions and approved the manuscript.

### Conflict of interest statement

The authors declare that the research was conducted in the absence of any commercial or financial relationships that could be construed as a potential conflict of interest.
